# Antibacterial activity of Cinnamomum verum and Thymus vulgaris essential oils on multidrug-resistant zoonotic bacteria isolated from dogs in southern Benin

**DOI:** 10.1099/acmi.0.000975.v3

**Published:** 2025-04-28

**Authors:** Ayaovi Bruno Yaovi, Arpita Das, Rama N. Behera, Paulin Azokpota, Souaïbou Farougou, Lamine Baba-Moussa, Franck Michels, Marie-Laure Fauconnier, Kiran Ambatipudi, Philippe Sessou

**Affiliations:** 1 Research Unit on Communicable Diseases, Polytechnic School of Abomey-Calavi, University of Abomey-Calavi, Abomey-Calavi, Benin; 2 Department of Biosciences and Bioengineering, Indian Institute of Technology Roorkee, Roorkee, Uttarakhand 247667, India; 3 Laboratory of Food Science and Technology, University of Abomey-Calavi, 03 B.P. 2819 Jericho-Cotonou, Benin; 4 Laboratory of Biochemistry and Molecular Typing in Microbiology, Department of Biochemistry and Cell Biology, Faculty of Science and Technology, University of Abomey-Calavi, Abomey-Calavi 05 BP 1604, Benin; 5 Laboratory of Chemistry of Natural Molecules, Gembloux Agro-Bio Tech, Liege University, Passage des Déportés 2, Gembloux 5030, Belgium

**Keywords:** Benin, *Cinnamomum verum*, *Escherichia coli*, essential oils, *Staphylococcus aureus*, *Thymus vulgaris*

## Abstract

Antibiotic resistance is a major public health problem. The search for new therapeutic alternatives is becoming urgent. Essential oils are a promising alternative. This study aimed to evaluate the antibacterial activities of essential oils from selected plants on multidrug-resistant zoonotic strains isolated from dogs. Essential oils from dried *Thymus vulgaris* leaves, *Cinnamomum verum* bark and *Cuminum cyminum* seeds were extracted and tested on five multidrug-resistant *Escherichia coli* and four *Staphylococcus aureus* isolated from dogs in southern Benin. The study showed that *T. vulgaris* essential oil was bacteriostatic, with an MIC equal to 2.5 µl ml^−1^ and a minimum bactericidal concentration (MBC) of 17 µl ml^−1^ for *E. coli* strains and 11.25 µl ml^−1^ for *S. aureus* strains. Regarding *C. verum* essential oil, its bacteriostatic power was characterized by an MIC of 1.25 µl ml^−1^ for the isolates tested and an average MBC of 11.50 µl ml^−1^ for *E. coli* and 12.19 µl ml^−1^ for *S. aureus*. On the other hand, *C. cyminum* essential oil was ineffective on the strains investigated. Additionally, *T. vulgaris* essential oil contained predominantly thymol (36.57%), p-cymene (30.51%) and carvacrol (7.62%), whilst *C. verum* essential oil contained cinnamaldehyde (88.76%). This study reveals the antibacterial activity of *T. vulgaris* dry leaf and *C. verum* bark essential oils on multi-resistant *E. coli* and *S. aureus* isolated from dogs. These two essential oils may be alternative candidates for combating antibiotic-resistant *E. coli* and *S. aureus* infections.

## Data Summary

The authors confirm all supporting data, code and protocols have been provided within the article.

## Introduction

Antibiotic resistance is a major threat to human and animal health. Indeed, the misuse of antibiotics in humans and animals has been identified as the main cause of the development of antibiotic-resistant pathogens [1]. This misuse includes the use of antibiotics (administration, dispensing or prescribing) for reasons other than treatment, non-completion of prescribed treatments and/or incorrect doses of antibiotics (under- or overdosing). The study conducted by O'Neill [2] showed that resistance to anti-infectives could be responsible for more than 10 million deaths a year, making it the leading cause by 2050, with an economic cost of US$100 billion if precautions are not taken.

Furthermore, close contact between pets and humans (petting, licking or physical injury), as well as the domestic environment, facilitates the transmission of antibiotic-resistant bacteria and genes [3]. Studies have reported antibiotic resistance in strains of *Staphylococcus aureus*, *Escherichia coli*, *Salmonella* spp., *Pseudomonas aeruginosa*, *Streptococcus pyogenes*, coagulase-negative *Staphylococcus* and *Staphylococcus pseudintermedius* isolated from dogs [4].

In Benin, multi-resistant strains of *E. coli* and *S. aureus* have been isolated from both free-ranging and caged dogs, posing a health threat to all those who come into contact with dogs harbouring these micro-organisms.

Nowadays, faced with the increase in drug-resistant bacteria and the limited availability of new, effective antibacterial agents, researchers are prompted to look for new strategies to replace or assist synthetic antibiotics [5]. Essential oils are known to possess a broad spectrum of bioactivities, including antimicrobial, anti-inflammatory, antioxidant, antiviral and antiproliferative properties [6,8]. The antimicrobial properties of essential oils have been known since antiquity and represent the most exploited to date. They can act as both bacteriostatic and bactericidal agents, being capable of inhibiting bacterial growth, thus blocking bacteria’s ability to reproduce and killing bacterial cells [5].

Given the consequences of infections caused by antimicrobial-resistant agents, there is an urgent need to look for alternatives to combat multi-resistant bacteria infections, which are emerging in dogs in our country. The present study aimed to evaluate the antibacterial activity of *Thymus vulgaris*, *Cinnamomum verum* and *Cuminum cyminum* essential oils on multi-resistant *E. coli* and *S. aureus* isolated from dogs in southern Benin.

## Methods

This study was approved by the Ethical Committee of Research Unit on Communicable Diseases (URMAT in French) of the Polytechnic School of Abomey-Calavi of the University of Abomey-Calavi (N°004/EPAC/LARBA/URMAT/CE/R).

### Plant samples used

*C. verum* bark (YH 1014/HNB), *C. cyminum* seeds (YH 1015/HNB) and dry leaves of *T. vulgaris* (YH 1016/HNB) purchased at the market and identified at the National Herbarium of Benin were used in this study.

### Essential oil extraction

Essential oils were extracted from dried *T. vulgaris* leaves, *C. verum* bark and *C. cyminum* seeds purchased at the market, at the ‘Laboratoire d’Etude et de Recherche en Chimie Appliquée’. The hydrodistillation method described by Kpatinvoh *et al*. [9] was used. One hundred grams of each plant material were introduced into 1 l of distilled water, and the mixture was distilled for 3 h in a Clevenger-type hydrodistillation apparatus. The essential oil obtained was separated from the aqueous phase, then dried with sodium sulphate (Na_2_SO_4_) and kept cool at 4 °C in a refrigerator for later use [8].

### Determination of inhibition diameters of essential oils

The antibacterial activities of essential oils from dry *T. vulgaris* leaves, *C. verum* bark and *C. cyminum* seeds were evaluated on five multi-resistant strains of *E. coli* (Ec01, Ec02, Ec03, Ec04 and Ec05) and four multi-resistant *S. aureus* strains (Sa01, Sa02, Sa03 and Sa04) isolated from dogs in southern Benin (Table 1). The disc diffusion method described by Sessou *et al*. [7] and slightly modified was used. A bacterial suspension was prepared by placing one colony in 5 ml of Mueller–Hinton broth (MHB) (Oxoid, Basingstoke, UK) and adjusted to 10^8^ c.f.u. ml^−1^. The inoculum thus prepared was inoculated onto Mueller–Hinton agar (Oxoid, Basingstoke, UK). Sterile Whatman paper discs (6 mm in diameter) coated with 10 µl of the essential oil were placed on the previously seeded agar. A negative control was prepared in the same way as the experimental test, and sterile distilled water was added in place of the essential oil. The experimental plates were incubated at 37 °C for 24 h. After incubation, the diameters of the zone of inhibition around the discs were measured. Essential oils with a diameter greater than 14 mm at 10 µl were selected for determination of MIC, MBC and antibiotic potency [7].

**Table 1.** Antibiotic susceptibility profile of multi-resistant *E. coli* and *S. aureus* strains studied

Bacteria Antibiotics	*E. coli*					*S. aureus*			
	Ec01	Ec02	Ec03	Ec04	Ec05	Sa01	Sa02	Sa03	Sa04
Penicillin G	R	R	R	R	R	R	R	R	R
Amoxicillin-clavulanic acid	S	S	S	S	I	S	S	S	S
Tetracycline	R	R	R	R	R	R	R	R	R
Gentamicin	S	S	S	S	S	S	S	S	S
Streptomycin	R	R	R	R	R	R	R	R	R
Erythromycin	–	–	–	–	–	R	S	R	S
Chloramphenicol	S	S	S	S	S	R	S	S	S
Ceftazidime	S	S	S	S	S	I	S	S	I
Cefotaxime	S	S	S	S	S	S	S	S	S
Cotrimoxazole	R	R	R	R	R	R	R	R	S

I, intermediate resistance; R, resistant to the antibiotic; S, susceptible to the antibiotic.

### Determination of the MIC and MBC of effective essential oils

The MIC and MBC of the essential oils effective on the bacteria tested were determined following the method described by Sessou *et al*. [7].

Concerning MIC, a stock solution of each essential oil was prepared from 2 ml of MHB, 40 µl of the essential oil and one drop of Tween 80. Thus, 100 µl of MHB was dispensed into each well of a 96-well microplate. One hundred microlitres of the oil stock solution were added to the first well, and successive dilutions of reason 2, well by well, were made up to the eleventh well in each row. All wells except those in the eleventh row were inoculated with 100 µl of bacterial suspension at 10^6^ c.f.u. ml^−1^, equal density on the McFarland scale. The microplate was then covered with parafilm and incubated at 37 °C for 18–24 h. The eleventh row represents the negative control, whilst the twelfth represents the positive control. The MIC is the lowest concentration for which no visible growth was noted. The MIC was considered to be the concentration of the well in which no visible bacterial growth was observed.

The MBC was determined successively to the MIC. Briefly, the contents of each well, ranging from the MIC value to the highest concentrations, were streaked onto the surface of Mueller–Hinton agar poured into Petri dishes. The wells of the positive and negative control rows were also streaked on the same agar to ensure the absence of bacterial growth in the wells of the negative control row and the presence of bacterial strains in the wells of the positive row. Inoculated agar plates were incubated at 37 °C for 24 h. On reading, MBC was the lowest extract concentration for which there were 0.01% surviving bacteria for the wells [7].

### Determining the antibiotic potency of essential oils

The antibiotic potency of the strain is determined by calculating the MBC/MIC ratio. When this ratio is less than or equal to 4, the essential oil is said to be bactericidal, and when the ratio is greater than 4, the essential oil is said to be bacteriostatic [10].

## Analysis of the constituents of essential oils effective against multi-resistant bacteria

### GC/MS

*T. vulgaris* leaves and *C. verum* bark essential oils were analysed on a Hewlett–Packard gas chromatograph model 7890, coupled to a Hewlett–Packard MS model 5875, equipped with a DB5 MS column (30 m×0.25 mm; 0.25 µm), programmed from 50 °C (5 min) to 300 °C at 5 °C min^−1^, with a 5 min hold. Helium was used as a carrier gas (1.0 ml min^−1^) with split mode injection (1 : 30); injector and detector temperatures were 250 °C and 280 °C, respectively. The mass spectrometer was operated in electron impact mode at 70 eV, with an electron multiplier (2500 eV) and an ion source temperature (180 °C); mass spectra data were acquired in scan mode in the m/z 33–450 range.

### GC/flame ionization detector

*T. vulgaris* leaves and *C. verum* bark essential oils were analysed on a Hewlett–Packard gas chromatograph model 6890, equipped with a DB5 MS column (30 m×0.25 mm; 0.25 µm), programmed from 50 °C (5 min) to 300 °C at 5 °C min^−1^, with a 5 min hold. Hydrogen was used as a carrier gas (1.0 ml min^−1^), with split mode injection (1 : 60); injector and detector temperatures were 280 °C and 300 °C, respectively. The essential oil was diluted in hexane: 1 out of 30. Compounds determined by GC in the various essential oils were identified by comparing their retention indices with those of reference compounds in the literature and confirmed by GC-MS by comparing their mass spectra with those of reference substances.

## Statistical analysis

Data from the analyses were entered into an Excel spreadsheet and analysed using R 4.3.1 software. Inhibition diameters were expressed as mean±sd. A one-factor ANOVA and an unpaired Student’s t-test were performed to compare the means of inhibition diameters. For a *P*-value<0.05, the difference between the means compared was statistically significant, and for a *P*-value>0.05, it was statistically non-significant.

## Results

### Antibacterial activity of the essential oils tested

Analysis of Table 2 shows that *E. coli* and *S. aureus* strains are sensitive to *T. vulgaris* and *C. verum* essential oils but are resistant to *C. cyminum* essential oil. The average diameter of the zone of inhibition of *T. vulgaris* was 31 and 36.5 mm, respectively, for *E. coli* and *S. aureus* strains, whilst that of *C. verum* was 33.8 and 35.25 mm, respectively, for the same strains.

**Table 2.** Average diameters of the inhibition zones of the essential oils of the plants studied

Strains	Code	*T. vulgaris*	*C. verum*	*C. cyminum*
		Mean	Mean	Mean
***E. coli***	Ec01	32	35	0
	Ec02	34	32	0
	Ec03	30	35	0
	Ec04	29	34	0
	Ec05	30	33	0
	Mean±sd	**31±2.00^ba^**	**33.8±1.30^ca^**	**0.00^aa^**
***S. aureus***	Sa01	27	35	10
	Sa02	44	35	13
	Sa03	32	37	8
	Sa04	43	34	13
	Mean±sd	**36.5±8.35^ba^**	**35.25±1.26^ba^**	**11±2.44^ab^**

Means followed by different letters are statistically different at the 5% threshold.

The first letters indicate comparisons of averages within the same bacterium, and the second letters indicate comparisons of means of the diameters of each oil between the two bacterial species studied.

### MIC and MBC of essential oils effective on multi-resistant strains

The MIC of *T. vulgaris* essential oil was the same for *E. coli* and *S. aureus* strains (2.5±0.00 µl ml^−1^), whilst the average MBC was 17 µl ml^−1^ for *E. coli* strains and 11.25 µl ml^−1^ for *S. aureus* strains. For *C. verum* essential oil, an MIC equal to 1.25 µl ml^−1^ was obtained for *E. coli* and *S. aureus* strains, whilst a mean MBC equal to 11.50 µl ml^−1^ was recorded for *E. coli* strains and 12.19 µl ml^−1^ for *S. aureus* strains (Table 3).

**Table 3.** MIC and MBC of *T. vulgaris* and *C. verum* essential oils

Strains	Code	*T. vulgaris*		*C. verum*	
		MIC (μl ml^−1^)	MBC (μl ml^−1^)	MIC (μl ml^−1^)	MBC (μl ml^−1^)
		Mean±sd	Mean±sd	Mean±sd	Mean±sd
***E. coli***	Ec01	2.5±00	20±00	1.25±00	10±00
	Ec02	2.5±00	5±00	1.25±00	2.5±00
	Ec03	2.5±00	20±00	1.25±00	15±00
	Ec04	2.5±00	20±00	1.25±00	15±00
	Ec05	2.5±00	20±00	1.25±00	15±00
	Mean±sd	2.5±00^a^	17±6.71^b^	1.25±00^a^	11.50±5.48^a^
***S. aureus***	Sa01	2.5±00	20±00	1.25±00	15±00
	Sa02	2.5±00	15±00	1.25±00	15±00
	Sa03	2.5±00	5±00	1.25±00	15±00
	Sa04	2.5±00	5±00	1.25±00	3.75±00
	Mean±sd	2.5±00^a^	11.25±7.5^a^	1.25±00^a^	12.19±5.62^a^

Means followed by different letters are statistically different at the 5% threshold.

### Antibiotic potency of *T. vulgaris* and *C. verum* essential oils effective on multi-resistant strains

Analysis of Table 4 shows that *T. vulgaris* and *C. verum* essential oils were bacteriostatic against the multi-resistant *E. coli* and *S. aureus* strains tested.

**Table 4.** Antibiotic power of *T. vulgaris* and *C. verum* essential oils

	*T. vulgaris*		*C. verum*	
**Strains**	MBC/CMI	Antibiotic potency	MBC/CMI	Antibiotic potency
***E. coli***	6.80	Bacteriostatic	9.20	Bacteriostatic
***S. aureus***	4.50	Bacteriostatic	9.75	Bacteriostatic

### Chemical composition of *T. vulgaris* and *C. verum* essential oils

Analysis of the essential oils revealed thymol (36.57%), p-cymene (30.51%) and carvacrol (7.62%) as the major compounds in *T. vulgaris* essential oil (Table 5). In *C. verum* essential oil, cinnamaldehyde was found to be the major component with a percentage of 88.76% (Table 6).

**Table 5.** Chemical composition of *T. vulgaris* essential oil

No.	Components	RT	RI	Percentage (%)
1	Camphene	3.5094	1074	0.72
2	d-Limonene	5.6248	1194	0.44
3	Eucalyptol	5.7768	1202	1.42
4	Gamma-Terpinene	6.6085	1240	0.43
5	p-Cymene	7.2039	1267	30.51
6	1-Octen-3-ol	11.5015	1444	1.42
7	(+)−2-Bornanone	12.8575	1498	0.48
8	Linalool	13.9004	1540	2.45
9	2-Isopropyl-5-methyl-anisole	14.9475	1582	3.08
10	Terpinen-4-ol	15.1333	1589	1.10
11	2-Isopropyl-1-methoxy-4-methylbenzene	15.205	1592	1.07
12	Estragole	16.7715	1656	0.82
13	Endo-borneol	17.4639	1685	2.18
14	γ-Cadinene	18.8446	1739	0.62
15	Anethole	20.6982	1810	1.65
16	p-Cymen-8-ol	21.3231	1833	0.51
17	Caryophyllene oxide	24.7769	1955	1.68
18	2-Propanone, 1-(4-methoxyphenyl)-	29.9196	2129	0.39
19	3-Allyl-6-methoxyphenol	30.2869	2142	2.52
20	Thymol	31.0891	2169	36.57
21	3-Methyl-4-isopropylphenol	31.5029	2183	1.15
22	Carvacrol	31.8027	2193	7.62
23	Eugenol acetate	32.9976	2234	1.16

RI, retention index; RT, retention time.

**Table 6.** Chemical composition of *C. verum* essential oil

No.	Components	RT	RI	Percentage (%)
1	Anethole	20.6984	1810	1.79
2	2-Propenal, 3-phenyl-	22.4084	1872	2.47
3	Cinnamaldehyde, trans	26.7362	2022	88.76
4	Cinnamyl acetate	29.8438	2127	3.20
5	Cinnamaldehyde, o-methoxy	37.914	2403	3.78

RI, retention index; RT, retention time.

## Discussion

This study assessed the antibacterial activity of *T. vulgaris*, *C. cyminum* and *C. verum* oils on multidrug-resistant *E. coli* and *S. aureus* isolated from dogs in southern Benin and determined the chemical composition of the effective essential oils.

The results of this study revealed the bacteriostatic power of *T. vulgaris* dry leaf and *C. verum* bark essential oils on multi-resistant *E. coli* and *S. aureus* strains.

The antimicrobial activity of *T. vulgaris* essential oil depends on the percentage composition of its main components [11]. In this study, thymol presented the highest percentage in the *T. vulgaris* essential oil tested (36.57%). This percentage may explain the bacteriostatic power of this oil. Indeed, thymol, chemically known as 2-isopropyl-5-methylphenol, is an edible monoterpene phenol found in abundance in certain plants such as *T. vulgaris* [12,13]. Studies investigating the mechanism of thymol’s antibacterial activity have indicated that its ability to integrate into the lipid layer of the cell membrane increases surface curvature. The hydrophilic part of the thymol interacts with the polar part of the membrane, whilst the hydrophobic benzene ring and aliphatic side chains sink into the inner part of the biological membrane [11]. This interaction leads to major changes in membrane structure, with destabilization of the lipid layer, reduced elasticity and increased fluidity. The change leads to increased permeability to potassium and hydrogen ions and also affects the activity of internal membrane proteins such as enzymes and receptors. After incorporation into the cell membrane, thymol interacts with the proteins embedded in it via a variety of non-specific mechanisms, leading to changes in the conformation and activity of internal and membrane proteins. As a result, cell membrane tension and destabilization can be induced by the presence of thymol [11].

The bacteriostatic power of *T. vulgaris* essential oil may also be linked to the presence of p-cymene in the oil tested. p-Cymene, known as p-cymol or p-isopropyltoluene, is an alkyl-substituted aromatic compound naturally present in the essential oils of various aromatic plants, including *Artemisia*, *Protium*, *Origanum* and *Thymus*. It is related to the terpene family, in particular the monocyclic monoterpenes [14]. Studies on the biological activities of this molecule have shown that it has synergistic antibacterial activity with other molecules such as carvacrol [15,16].

Carvacrol is a natural monoterpene phenol that is particularly abundant in the essential oils of *Origanum vulgare*, *T. vulgaris*, *Lepidium flavum*, *Citrus aurantium bergamia* and other plants [17,18]. Carvacrol interacts with the cell membrane via hydrogen bonding, making membranes and mitochondria more permeable and disintegrating the outer cell membrane [19]. Its antibacterial activity on *E. coli* [20] and methicillin-resistant *S. aureus* (ATCC-33591) strains has been demonstrated [21]. The bacteriostatic power of *T. vulgaris* essential oil can also be attributed to the synergistic activity of thymol, p-cymene and carvacrol [6,22, 23].

*C. verum* essential oil’s bacteriostatic power against *E. coli* and *S. aureus* strains can be linked to its high cinnamaldehyde content. Cinnamaldehyde is a chemical compound that occurs naturally as a trans-stereoisomer, namely, (2E)-3-phenylprop-2-enal or trans-cinnamaldehyde, which is particularly abundant in the essential oils of plants in the *Cinnamomum* genus [5]. Trans-cinnamaldehyde has been shown to possess substantial antimicrobial activity, as well as a range of medicinal properties [24,26]. The antibacterial activities of this molecule extend to a range of Gram-positive and negative bacteria, such as *E. coli*, *Bacillus subtilis*, *Staphylococcus* spp., *Listeria* spp., *Salmonella* spp., *Lactobacillus sakei*, *Campylobacter jejuni*, *Vibrio* spp., *Pseudomonas* spp., *Porphyromonas gingivalis*, *S. pyogenes* and *Cronobacter sakazakii* [27].

In this study, the essential oil of *C. cyminum* seeds proved ineffective on the multidrug-resistant *E. coli* and *S. aureus* strains investigated. However, in the study by Sharifi *et al*. [28] *C. cyminum* seed oil showed bacteriostatic and bactericidal activities on multidrug-resistant *S. aureus* isolated from patients and from the milk of cattle suffering from mastitis. This difference in results is linked to the different sources of bacteria studied, the resistance mechanisms developed by these strains and the chemical composition of the oils tested.

## Conclusion

This study demonstrated the antibacterial activity of *T. vulgaris* dry leaf and *C. verum* bark essential oils on multi-resistant *E. coli* and *S. aureus* strains. Both oils are bacteriostatic on the zoonotic agents studied. The study also revealed the components responsible for antibacterial activity. Thymol, p-cymene and carvacrol were the main components of *T. vulgaris* essential oil, whilst cinnamaldehyde was the main component of *C. verum* essential oil. The two oils investigated in this study may be alternative candidates for combating infections with resistant bacterial agents, such as *E. coli* and *S. aureus*.

## Abbreviations

MBC: minimum bactericidal concentration
MHB: Mueller–Hinton broth

## References

[1] Mugwaneza D, Rwagasore E, El-Khatib Z, Dukuziyaturemye P, Omolo J (2024). Factors associated with inappropriate use of antibiotics among animal health professionals in selected districts of Rwanda, 2021. J Epidemiol Glob Health.

[2] O’Neill J (2016). Tackling drug-resistant infections globally: final report and recommendations (report).

[3] Caneschi A, Bardhi A, Barbarossa A, Zaghini A (2023). The use of antibiotics and antimicrobial resistance in veterinary medicine, a complex phenomenon: a narrative review. Antibiotics.

[4] Yaovi AB, Sessou P, Tonouhewa ABN, Hounmanou GYM, Thomson D (2022). Prevalence of antibiotic-resistant bacteria amongst dogs in Africa: a meta-analysis review. Onderstepoort J Vet Res.

[5] Usai F, Di Sotto A (2023). Trans-cinnamaldehyde as a novel candidate to overcome bacterial resistance: an overview of in vitro studies. Antibiotics.

[6] Micucci M, Protti M, Aldini R, Frosini M, Corazza I (2020). *Thymus vulgaris* L. essential oil solid formulation: chemical profile and spasmolytic and antimicrobial effects. Biomolecules.

[7] Sessou P, Yaovi BA, Yovo M, Gamedjo J, Dossa F (2018). Phytochemistry and antibacterial activity of plants extracts compared with two commercial antibiotics against *E coli* responsible for avian colibacillosis in Benin. IJPM.

[8] Sharifi-Rad J, Dey A, Koirala N, Shaheen S, El Omari N (2021). *Cinnamomum* species: bridging phytochemistry knowledge, pharmacological properties and toxicological safety for health benefits. Front Pharmacol.

[9] Kpatinvoh B, Adjou ES, Dahouenon-Ahoussi E, Konfo TRC, Atrevi B (2017). Efficacité des huiles essentielles de trois plantes aromatiques contre la mycoflore d’altération du niéBé (Vigna unguiculata L., Walp) collecté dans les magasins de vente du Sud-Bénin. J App Bioscience.

[10] Mamadou R, Moussa I, Philippe S, Yehouenou B, Agbangnan D (2014). Etude phytochimique, activités antiradicalaire, antibactérienne et antifongique d’extraits de sebastiania chamaelea (L.) Müll.arg. JSOAC.

[11] Kowalczyk A, Przychodna M, Sopata S, Bodalska A, Fecka I (2020). Thymol and thyme essential oil-new insights into selected therapeutic applications. Molecules.

[12] Amiri H (2012). Essential oils composition and antioxidant properties of three thymus species. Evid Based Complement Alternat Med.

[13] Nagoor Meeran MF, Javed H, Al Taee H, Azimullah S, Ojha SK (2017). Pharmacological properties and molecular mechanisms of thymol: prospects for Its therapeutic potential and pharmaceutical development. Front Pharmacol.

[14] Balahbib A, El Omari N, Hachlafi NEL, Lakhdar F, El Menyiy N (2021). Health beneficial and pharmacological properties of p-cymene. Food Chem Toxicol.

[15] Ersanli C, Tzora A, Skoufos I, Fotou K, Maloupa E (2023). The assessment of antimicrobial and anti-biofilm activity of essential oils against *Staphylococcus aureus* strains. Antibiotics.

[16] Rattanachaikunsopon P, Phumkhachorn P (2010). Assessment of factors influencing antimicrobial activity of carvacrol and cymene against Vibrio cholerae in food. J Biosci Bioeng.

[17] Marinelli L, Fornasari E, Eusepi P, Ciulla M, Genovese S (2019). Carvacrol prodrugs as novel antimicrobial agents. Eur J Med Chem.

[18] Sharifi-Rad M, Varoni EM, Iriti M, Martorell M, Setzer WN (2018). Carvacrol and human health: a comprehensive review. Phytother Res.

[19] Imran M, Aslam M, Alsagaby SA, Saeed F, Ahmad I (2022). Therapeutic application of carvacrol: a comprehensive review. Food Sci Nutr.

[20] Khan I, Bahuguna A, Shukla S, Aziz F, Chauhan AK (2020). Antimicrobial potential of the food-grade additive carvacrol against uropathogenic *E. coli* based on membrane depolarization, reactive oxygen species generation, and molecular docking analysis. Microb Pathog.

[21] Selvaraj A, Valliammai A, Muthuramalingam P, Priya A, Suba M (2020). Carvacrol targets SarA and CrtM of methicillin-resistant *Staphylococcus aureus* to mitigate biofilm formation and Staphyloxanthin synthesis: an in vitro and in vivo approach. ACS Omega.

[22] Andrade-Ochoa S, Nevárez-Moorillón GV, Sánchez-Torres LE, Villanueva-García M, Sánchez-Ramírez BE (2015). Quantitative structure-activity relationship of molecules constituent of different essential oils with antimycobacterial activity against Mycobacterium tuberculosis and Mycobacterium bovis. BMC Complement Altern Med.

[23] Kachur K, Suntres Z (2020). The antibacterial properties of phenolic isomers, carvacrol and thymol. Crit Rev Food Sci Nutr.

[24] Ahmed HM, Ramadhani AM, Erwa IY, Ishag OAO, Saeed MB (2020). Phytochemical screening, chemical composition and antimicrobial activity of cinnamon verum bark. IRJPAC.

[25] Vazirian M, Alehabib S, Jamalifar H, Fazeli R, Toosi A (2015). Antimicrobial effect of cinnamon (cinnamomum verum J. presl) bark essential oil in cream-filled cakes and pastries. Res J Pharmacogn.

[26] Zhu R, Liu H, Liu C, Wang L, Ma R (2017). Cinnamaldehyde in diabetes: a review of pharmacology, pharmacokinetics and safety. Pharmacol Res.

[27] Doyle AA, Stephens JC (2019). A review of cinnamaldehyde and its derivatives as antibacterial agents. Fitoterapia.

[28] Sharifi A, Mohammadzadeh A, Salehi TZ, Mahmoodi P, Nourian A (2021). Cuminum cyminum L. Essential Oil: a promising antibacterial and antivirulence agent against multidrug-resistant *Staphylococcus aureus*. Front Microbiol.

